# Life-Cycle Diphenyl Phosphate (DPhP) Exposure Reveals Sex-Differential Functional Alterations in the Zebrafish Gut Microbiota

**DOI:** 10.3390/biology15141192

**Published:** 2026-07-19

**Authors:** Tong Zhou, Ruiqi He, Ya Li, Yuwei Xie, Linyan Deng, Meng Xia, Xiaolong Lian, Jingjing An, Yufeng Gong, Qiliang Chen

**Affiliations:** 1 Chongqing Key Laboratory of Conservation and Utilization of Freshwater Fishes, Animal Biology Key Laboratory of Chongqing Education Commission of China, Chongqing Normal University, Chongqing 401331, China; a19923011672@163.com (T.Z.); rqhe001@163.com (R.H.); yali010708@163.com (Y.L.); 13272769369@163.com (L.D.); xiam2002@163.com (M.X.); annjingjing947@163.com (J.A.); 2 Nanjing Institute of Environmental Sciences, Ministry of Ecology and Environment, Nanjing 210042, China; xieyuwei@nies.org; 3 Chongqing International Institute for Immunology, Chongqing 401338, China; lianxl369@163.com; 4 College of Marine Life Sciences, Ocean University of China, Qingdao 266003, China; yufeng.gong@ouc.edu.cn

**Keywords:** diphenyl phosphate, zebrafish, gut microbiota, sex differences, functional prediction, 16S rRNA gene sequencing

## Abstract

Fish gut microbiota is essential for maintaining host physiological health but is constantly threatened by environmental chemicals. Diphenyl phosphate (DPhP), a major degradation product of organophosphate flame retardants and an environmental pollutant widely detected in aquatic ecosystems, is a chemical of emerging concern; however, few studies have addressed its effects on the gut microbiota. In this study, zebrafish were exposed to environmentally relevant concentrations of DPhP (0.8, 3.9, or 35.6 μg/L) from the embryonic stage to adulthood over 120 days. Using V3-V4 region 16S rRNA gene sequencing and functional gene prediction, we systematically analyzed the sex-specific responses of the gut microbiota. The results showed that life-cycle DPhP exposure exerts markedly different effects on the gut microbiota of male and female zebrafish. In females, DPhP induced concentration-dependent, stepwise Tax4Fun2 predicted functional shifts suggestive of compensation: at the low concentration (0.8 μg/L), energy metabolism and cofactor/vitamin metabolism functions were predicted to be enhanced; at the medium concentration (3.9 μg/L), Fusobacteria (mainly *Cetobacterium ceti*) became dominant, accompanied by structural remodeling; at the high concentration (35.6 μg/L), genetic information processing and amino acid biosynthesis pathways were upregulated, suggesting active substance synthesis. In contrast, the gut microbiota of male zebrafish did not exhibit significant changes in diversity but showed widespread predicted functional impairment. Core energy metabolism and cofactor/vitamin metabolism functions were significantly suppressed, while taxa associated with pro-inflammatory phyla (Proteobacteria, Verrucomicrobia) expanded. Functional compensation in males was limited to xenobiotic degradation and fatty acid metabolism, suggesting a potential pathological shift. These findings suggest a sex-differential response of DPhP from the perspective of the gut microbiota, providing new evidence for the environmental risk assessment of organophosphorus flame retardants.

## Simple Summary

Flame retardants are added to many everyday products. Their breakdown products often end up in rivers and lakes. One such product is diphenyl phosphate (DPhP). This chemical can harm fish, but it is unknown whether it affects male and female fish differently by altering their gut bacteria. In this study, we exposed zebrafish from embryos to adults for 120 days to water containing low, medium, or high concentrations of DPhP, and then examined their gut bacteria. The chemical caused markedly different changes in the two sexes. In females, the gut bacteria responded in a dose-dependent manner: at low levels, they predicted functions for energy and vitamins; at medium levels, a beneficial bacterium became dominant; at high levels, production of anabolic precursors was enhanced. In males, the gut bacteria exhibited predicted reductions in energy- and vitamin-synthesis capacity, while bacteria previously associated with inflammation increased in relative abundance. These results show that a common pollutant can affect male and female fish differently. This knowledge helps us better assess risks to aquatic life and protect aquatic health.

## 1. Introduction

As pivotal vertebrates in aquatic environments, fish harbor vast and complex bacterial communities in their guts, which are collectively referred to as the fish gut microbiota [1,2,3]. The gut microbiota has co-evolved with vertebrate hosts for millions of years, playing a critical role in nutrient digestion, immune regulation, and health maintenance [3]. However, this sensitive microecosystem is continuously challenged by environmental pollutants [4,5,6]. The gradual phase-out of legacy brominated flame retardants has led to the increasing environmental accumulation of their substitutes, organophosphate ester flame retardants (OPFRs) [7,8,9]. These substances enter organisms through multiple pathways, like diet, respiration, and skin contact [10,11], ultimately interacting closely with the gut microbiota.

Among these, diphenyl phosphate (DPhP), a representative organophosphorus compound, is ubiquitous in the environment [12]. On one hand, it can be used as a flame retardant, plasticizer, or material intermediate [12]. On the other hand, and more importantly, it is a major degradation and metabolic product of some common organophosphorus flame retardants (e.g., triphenyl phosphate, TPhP) in organisms [12,13]. This implies that even without direct contact, organisms can be exposed to DPhP through the biotransformation of its parent compounds [13]. DPhP carries potential risks of bioaccumulation and biomagnification, exhibits biotoxic effects [12], and is widely recognized as a significant emerging environmental pollutant. In wastewater and industrial effluents, concentrations have been reported up to the μg/L range [14,15]. Currently, toxicity studies have mostly focused on its parent compound, TPhP, while research on DPhP itself remains limited. For example, early-life exposure to TPhP significantly disrupts the composition of the mouse gut microbiota and impairs its metabolic function [16]. TPhP induces gut dysbiosis at multiple taxonomic levels [17], reduces the abundance of anti-inflammatory probiotics, and increases pro-inflammatory bacteria [18]. In addition, TPhP alters the gut microbiota composition of aquatic turtles, increasing the relative abundance of certain pathogenic bacteria [19]. However, as a direct exposure agent and major metabolic end product, the independent biotoxicity of DPhP to the gut microbiota remains largely understudied. Given that the physicochemical properties, biotransformation pathways, and target organ distributions of the parent compound and its metabolite may differ significantly, directly extrapolating the toxicity of TPhP to the risk of DPhP introduces substantial uncertainty.

Previous studies have shown that a variety of environmental pollutants (e.g., organophosphate flame retardants, fluoride, air pollutants, and triphenyltin) exert significant sex-specific effects on the gut microbiota. For example, tris(1,3-dichloro-2-propyl) phosphate (TDCIPP) exerts sex-specific effects on the zebrafish gut microbiota. Males alleviate intestinal damage by increasing alpha diversity, whereas females show abnormally elevated abundance of pathogenic bacteria, which impairs intestinal barrier function and retards growth [20]. TPhP disrupts the gut microbiota of dams and their offspring, with male offspring showing the most pronounced effects [21]. Fluoride-induced gut dysbiosis is particularly evident in male mice [22]. Air pollutants (e.g., black carbon, PM_2.5_) also exert stronger negative impacts on microbial diversity in boys than in girls [23]. Furthermore, triphenyltin exposure not only induces sex-specific gut microbiota dysbiosis in marine medaka but also exhibits sex-specific transgenerational effects on offspring gut health [24]. The potential mechanisms underlying these sex-related differences include direct regulation of the gut microecosystem by sex hormones (estrogens and androgens) [25], sexually dimorphic expression of drug-metabolizing enzymes (e.g., cytochrome P450) in the liver and intestine [26], and sex-specific immune responses [27]. Notably, DPhP and its parent compound TPhP have been reported to possess weak estrogenic or anti-androgenic activities [28,29], suggesting that they may differentially shape the gut microbiota of males and females by interfering with sex hormone signaling pathways. Nevertheless, whether the effects of DPhP on the gut microbiota differ between males and females remains largely unexplored.

Our previous study found that life-cycle exposure to DPhP at concentrations of 0.8–35.6 μg/L significantly reduced body mass and length only in male zebrafish, with no observable effect on females; multi-omics analysis further revealed that DPhP suppressed hepatic oxidative phosphorylation and fatty acid oxidation in males, leading to reduced energy output, while female livers exhibited adaptive upregulation of these pathways [30]. Given the well-established role of the gut microbiota in host energy metabolism and its interaction with the liver via the gut–liver axis, it remains unclear whether DPhP exposure also induces sex-specific remodeling of the gut microbiota, and whether such microbial changes contribute to the observed growth inhibition. We therefore hypothesized that DPhP exposure induces sex-specific remodeling of the gut microbiota, which may underlie the sex-differential growth inhibition.

To test this hypothesis, the present study used zebrafish as a model, which has well-defined sex differentiation and a clear sexual maturation window [31], making it ideal for characterizing the sex-specific intestinal toxicity of pollutants. Zebrafish also have a short generation time, and their gut microbiota has been extensively characterized in previous studies [32], providing a robust reference for this investigation. Zebrafish were exposed to DPhP at concentrations of 0.8, 3.9, or 35.6 μg/L from the embryonic stage to adulthood (120 days). After exposure, intestinal tissues were collected for 16S rRNA gene sequencing of the V3-V4 region. To our knowledge, this is the first study to investigate the sex-specific effects of DPhP on the gut microbiota of fish under environmentally relevant concentrations across a full life-cycle exposure scenario, and aims to provide a scientific basis for the health risk assessment of DPhP exposure.

## 2. Materials and Methods

### 2.1. Compounds and Reagents

DPhP (99% purity, CAS No. 838-85-7) was purchased from Shanghai Aladdin Biochemical Technology Co., Ltd. (Shanghai, China). Tricaine methanesulfonate (MS-222) was obtained from Sigma-Aldrich (St. Louis, MO, USA).

### 2.2. Zebrafish Husbandry

Adult zebrafish (AB strain, 4 months old) were obtained from the National Zebrafish Resource Center (Wuhan, China). Prior to experimentation, males and females were acclimatized separately for two weeks in a recirculating aquaculture system under controlled conditions (14 h: 10 h light/dark cycle, 28 °C) and fed three times daily with a combination of commercial feed and *Artemia nauplii*. The experiment was approved by the Key Laboratory of Animal Biology of Chongqing at Chongqing Normal University (Permit No. Zhao-20191226-01).

### 2.3. Exposure and Sample Collection

The zebrafish exposure design and feeding protocols were the same as those described previously [30]. Briefly, for embryo collection, males and females (female/male ratio = 1:2) were placed in spawning boxes overnight. After spawning, normal embryos were randomly distributed into 16 tanks (20 cm × 14 cm × 8 cm) at a density of 150 embryos per tank. Each tank contained 1 L of aqueous solution spiked with DPhP at nominal concentrations of 0 (Control), 1 (Low), 10 (Medium), or 100 (High) μg/L, with four replicate tanks per concentration. The measured concentrations of DPhP were below the limit of detection for the control group, and were 0.8 ± 0.2, 3.9 ± 1.0, and 35.6 ± 9.9 μg/L for the nominal concentrations of 1, 10, and 100 μg/L, respectively. Measured concentrations are expressed as mean ± SEM. The High group exhibited greater variation, which may be attributable to microbial degradation and metabolism of DPhP by adult fish during the 48 h renewal cycle. Similar discrepancies between nominal and measured concentrations have also been reported previously for other organophosphate esters in semi-static exposure systems [30].

Exposure tanks containing embryos were placed in an incubator under controlled conditions (14 h: 10 h light/dark cycle, 28 °C) for 20 days. At 21 days post fertilization (dpf), larvae were transferred to an indoor semi-static system and maintained under the same photoperiod and temperature (27 ± 0.3 °C) for continuous exposure until 120 dpf. During the exposure, half of the water was renewed every two days. To accommodate growth, fish were gradually moved to larger tanks to maintain appropriate stocking densities as follows: approximately 120, 60, 10, and 5 fish per liter at 6–15 dpf, 16–30 dpf, 31–60 dpf, and 61–120 dpf, respectively. Feeding regimes were adjusted according to developmental stage: live *Paramecia* from 6 to 20 dpf; a mixture of live *Paramecia* and *Artemia* from 21 to 50 dpf; and a combination of live *Artemia* and commercial pellet diet from 51 to 120 dpf, all provided three times daily to apparent satiation. Water quality was monitored weekly and remained stable: temperature 27 ± 0.3 °C, pH 7.7 ± 0.1, and dissolved oxygen > 6 mg/L.

After DPhP exposure, zebrafish were fasted for 24 h, anesthetized with 0.01% MS-222, and sex was confirmed by dissecting and visually inspecting the gonads at the time of sample collection, which is a standard method for sex determination in adult zebrafish. The body surface was wiped with gauze soaked in 75% ethanol. An incision was made along the abdomen with a scalpel, and the skin and muscle were removed to expose the coelomic cavity. The entire intestine was immediately isolated, quickly placed into a sterile and RNase-free centrifuge tube, sealed, frozen in liquid nitrogen, and stored at −80 °C. The intestinal tracts from 10 fish per tank were pooled into one sample to obtain sufficient DNA for sequencing and to reduce inter-individual variation that could obscure treatment-related effects and were sent to Beijing Biomarker Technologies Co., Ltd. (Beijing, China) for sequencing.

### 2.4. Gut Microbiome Metabarcoding

Gut microbiome was amplified using primer pair of F (5′-ACTCCTACGGGAGGCAGCA-3′) and R (5′-GGACTACHVGGGTWTCTAAT-3′) targeting V3-V4 region of the bacterial 16S rRNA (length < 490 bp). Negative controls (sterile water) were included during DNA extraction and PCR amplification to monitor potential contamination. PCR products were purified, quantified, and sequenced on the Illumina HiSeq 2500 platform. Paired-end raw reads were assembled using FLASH v1.2.11 [33] and checked by use of Trimmomatic v0.33 [34]. ZOTUs (Zero-radius OTUs) were denoised using the UNOISE3 algorithm in USEARCH v11 [35,36], and chimeras were filtered by UCHIME v4.2 [37]. Taxonomy of the filtered ZOTUs were annotated against the GSR-DB database [38] with a confidence threshold greater than 0.8 in QIIME2 environment [39].

### 2.5. Statistical Analysis

Potential contaminating sequences, including those taxonomically assigned to chloroplasts, mitochondria, and phages, were identified and removed based on reference database classification prior to downstream analyses. Statistics were conducted under PRIMER 7 software and R (v4.5.1) environment. To correct for uneven sequencing depth across samples, all samples were rarefied to 18,807 sequences per sample. Alpha diversity indices (Shannon, Observed features, Pielou, Faith PD), and beta diversity (Weighted UniFrac) distance matrices were calculated using microeco package [40]. Differential alpha diversity indices among groups were tested by use of Kruskal–Wallis test. Principal Coordinates Analysis (PCoA) based on Weighted UniFrac distances was used to visualize beta diversity, and Permutational Multivariate Analysis of Variance test (PERMANOVA, permutation runs = 999) was employed to assess the significance of differences in community structure between groups. Differences in gut microbiota composition between groups were tested using ANOVA. Linear discriminant analysis Effect Size (LEfSe) analysis and Random Forests were used to identify biomarkers showing significant differences between groups. Specifically, LEfSe analysis quantified the effect sizes of differential taxa via LDA score bar plots and illustrated their phylogenetic relationships and taxonomic characteristics across multiple taxonomic levels using cladograms. Random Forest analysis was employed to identify the taxa at the class and genus levels that served as important contributors to intergroup discrimination. Microbial functions were predicted using Tax4Fun2 package [41], and ANOVA was used to test for differences between groups.

## 3. Results

### 3.1. Sex Differential Gut Microbiome of Zebrafish

**Figure 1:**
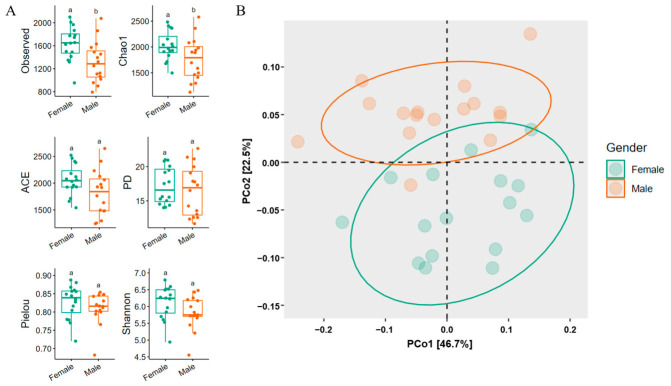
(**A**) Alpha diversity of the gut microbiota in female and male zebrafish. Different letters indicate significant differences between groups (*p* < 0.05), whereas the same letters indicate no statistically significant difference. (**B**) Weighted UniFrac distance-based beta diversity analysis. PCoA plot showing the community structure variation in the gut microbiota between female and male zebrafish across all exposure groups.

Alpha diversity comparisons between the female and male groups, which were pooled across all treatment groups to provide a global view of sex-associated differences under the complete exposure context, revealed that the Observed features index (*p* = 0.004) and Chao1 index (*p* = 0.04) were significantly higher in females than in males, while the Shannon index, Pielou index, and Faith PD index showed no significant differences (Figure 1A). PERMANOVA based on Weighted UniFrac distances revealed a highly significant effect of host sex on the overall gut microbial community structure (*F* = 6.640, *p* = 0.001) (Figure 1B).

After quality control and paired-end merging, 1,516,892 clean reads were generated from 32 samples, with per-sample depths ranging from 18,807 to 65,669 reads (mean ± SD: 47,402.88 ± 14,123). The mean sequencing depth for each treatment group was: Control, 37,165.75 ± 7652.50 (females) and 60,747.50 ± 1683.18 (males); Low, 24,399.25 ± 5169.29 (females) and 62,507.25 ± 2969.97 (males); Medium, 52,744.00 ± 9875.88 (females) and 48,029.75 ± 11,105.96 (males); High, 40,707.25 ± 13,594.97 (females) and 52,922.25 ± 5707.41 (males). A total of 5460 ZOTUs were obtained.

### 3.2. Effects of DPhP Exposure on the Female Gut Microbiota of Zebrafish

#### 3.2.1. Effects of DPhP Exposure on Gut Microbiota Diversity in Female Zebrafish

**Figure 2:**
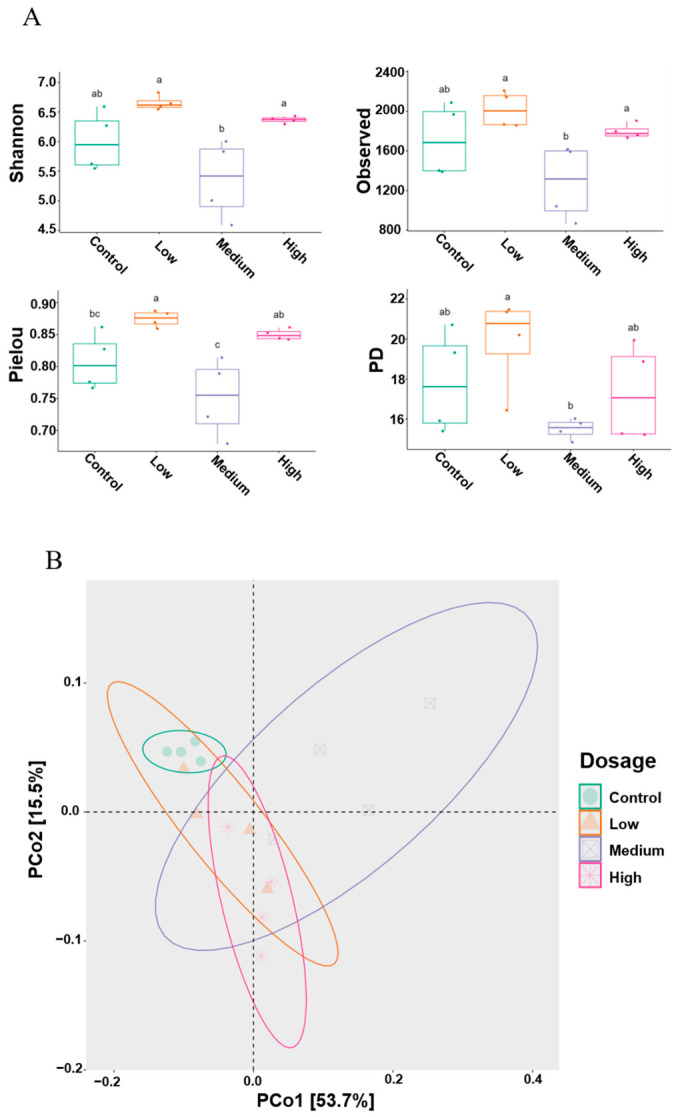
Effects of DPhP exposure on gut microbiota diversity in female zebrafish. (**A**) Alpha diversity indices (Shannon, Observed features, Pielou evenness, and Faith PD) in the control group and under different concentrations of DPhP. Different letters indicate significant differences between groups (*p* < 0.05), whereas the same letters indicate no statistically significant difference. (**B**) Weighted UniFrac distance-based beta diversity analysis. PCoA plot showing the community structure variation in the gut microbiota in female zebrafish across the control and different DPhP exposure groups.

DPhP exposure exerted a significant influence on the alpha diversity of the gut microbiota in female zebrafish. Overall comparisons among groups revealed statistically significant differences in the Shannon index (*p* = 0.003), Observed features index (*p* = 0.02) and Pielou evenness index (*p* = 0.004). Across the four alpha diversity metrics assessed, the highest values of all four indices were observed in the Low group, whereas the lowest values were concentrated in the Medium group (Figure 2A). Further pairwise comparisons showed that the Pielou index was significantly higher in the Low group than in the Control group (*p* = 0.03). For the Shannon, Observed features, and Faith PD index, no significant differences were detected between any treatment group and the Control group.

Beta diversity analysis further revealed a marked shift in the gut microbial community structure of female zebrafish following DPhP exposure. PCoA demonstrated that samples from the Control group and the Medium group were clearly separated (Figure 2B) and PERMANOVA confirmed that the difference was statistically significant (*F* = 7.64, *p* = 0.03).

#### 3.2.2. Alterations in Gut Microbiota Composition and Enrichment of Group-Specific Biomarkers in Female Zebrafish Under DPhP Exposure

**Figure 3:**
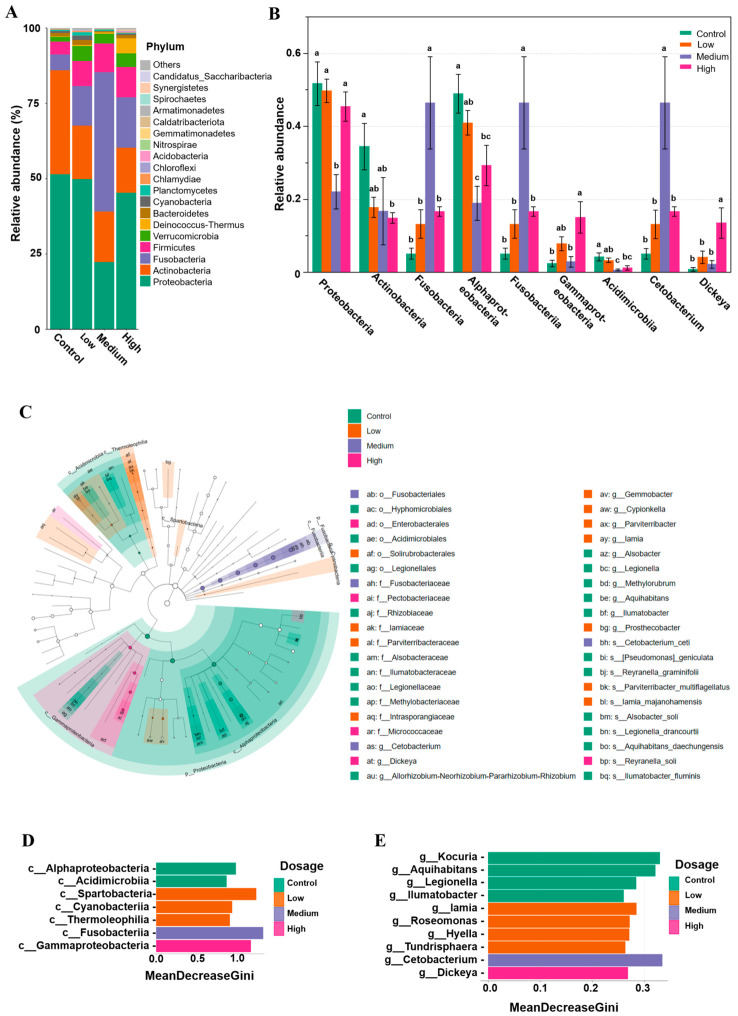
Alterations in gut microbiota composition and enrichment of group-specific biomarkers in female zebrafish under DPhP exposure. (**A**) Relative abundance of gut microbiota at the phylum level in each experimental group. (**B**) Differential relative abundance of gut microbiota at the phylum, class, and genus levels in each experimental group. Different letters indicate significant differences between groups (*p* < 0.05), whereas the same letters indicate no statistically significant difference. (**C**) LEfSe cladogram of the gut microbiota in female zebrafish. Circles radiating from the inside to the outside represent taxonomic levels from kingdom to species. Nodes in different colors indicate taxa significantly enriched in the group corresponding to that color; colorless nodes indicate taxa with no significant differences between groups. Node diameter is proportional to the relative abundance of the taxon. Major taxonomic levels such as phylum and class are labeled directly on the diagram, while other levels are marked with letters, and their specific names are provided in the legend on the right. (**D**) Random forest analysis of the gut microbiota at the class level in each experimental group. (**E**) Random forest analysis of the gut microbiota at the genus level in each experimental group.

Phylum-level relative abundance analysis revealed that Proteobacteria, Actinobacteria, Fusobacteria, Firmicutes, and Verrucomicrobia were the dominant phyla in the gut microbiota across all experimental groups, collectively accounting for over 90% of the total relative abundance (Figure 3A). Notably, in addition to these five phyla, Deinococcus–Thermus was also identified as a dominant phylum in the High group. Regarding the ranking of relative abundances, the Control and Low groups showed a consistent descending order: Proteobacteria > Actinobacteria > Fusobacteria > Firmicutes > Verrucomicrobia. The descending order in the Medium group was Fusobacteria > Proteobacteria > Actinobacteria > Firmicutes > Verrucomicrobia, whereas that in the High group was Proteobacteria > Fusobacteria > Actinobacteria > Firmicutes > Deinococcus–Thermus > Verrucomicrobia. These results indicate a marked difference in taxonomic composition between the Control and Medium groups, as well as a clear difference between the Control and High groups. One-way ANOVA further confirmed that, compared with the Control group, the relative abundance of Proteobacteria was significantly decreased in the Medium group (*p* = 0.004), whereas that of Fusobacteria was significantly increased (*p* = 0.005). In addition, the relative abundance of Actinobacteria in the High group was significantly lower than that in the Control group (*p* = 0.03) (Figure 3B).

Combined LEfSe (Figure 3C) and random forest (Figure 3D,E) analyses identified Alphaproteobacteria and Acidimicrobiia as the key biomarkers in the Control group. ANOVA (Figure 3B) revealed that the relative abundances of Alphaproteobacteria and Acidimicrobiia were significantly higher in the Control group than in the Medium (*p* = 0.005 and 0.004, respectively) and High groups (*p* = 0.01 and 0.01, respectively). Furthermore, the LEfSe cladogram showed significant enrichment of the phylum Proteobacteria, the class Alphaproteobacteria, the order Hyphomicrobiales, and its subordinate taxa in the Control group. In the Low group, neither LEfSe analysis based on LDA scores nor random forest analysis identified any shared characteristic taxa. Although both methods detected some overlapping gut microbiota, the relative abundances of these overlapping taxa were low, in sharp contrast to the high-abundance characteristic microbiota found in the other groups. For the Medium group, LEfSe analysis revealed significant enrichment of a taxonomic lineage spanning from Fusobacteria to the species *Cetobacterium ceti*. Random forest analysis similarly confirmed that Fusobacteriia and *Cetobacterium* were important contributors in the Medium group, and ANOVA showed that the relative abundances of these two taxa were significantly higher in the Medium group than in the Control group (*p* = 0.005 for both). For the High group, LEfSe analysis identified pronounced enrichment from Gammaproteobacteria to the genus *Dickeya*. Random forest analysis corroborated the high contributions of Gammaproteobacteria and *Dickeya*, and ANOVA demonstrated that the relative abundances of both taxa were significantly increased in the High group compared with the Control group (*p* = 0.02 and *p* = 0.01, respectively).

#### 3.2.3. KEGG Functional Analysis of Gut Microbiota in Female Zebrafish Under DPhP Exposure

**Figure 4:**
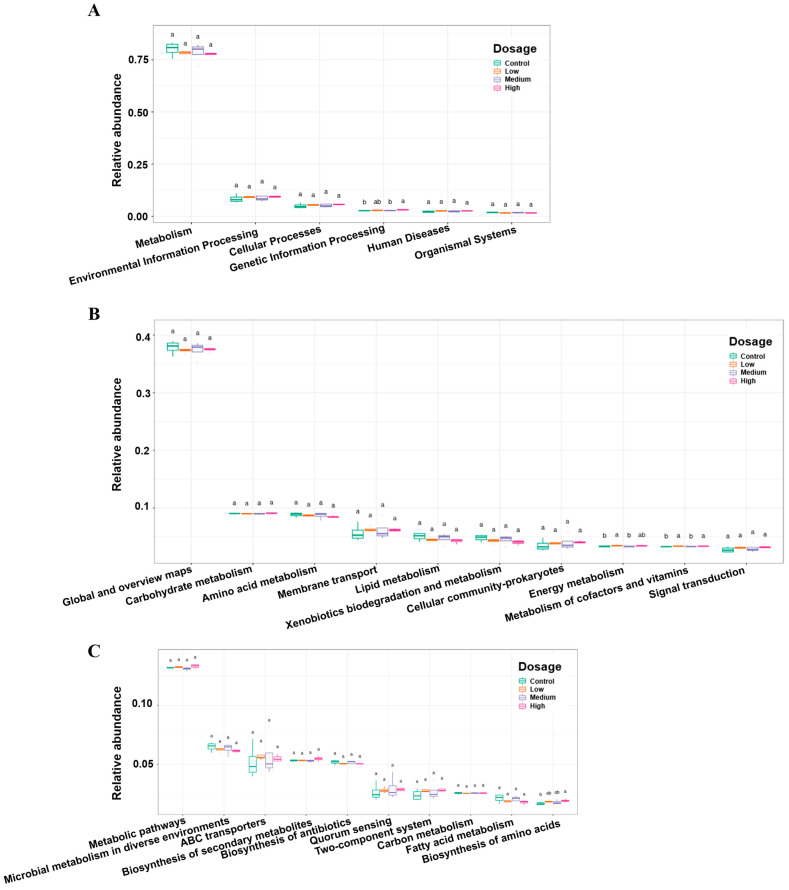
Differential relative abundance of KEGG functional genes in the gut microbiota of female zebrafish under DPhP exposure. (**A**) Level 1 functional categories. (**B**) Level 2 functional subcategories. (**C**) Level 3 functional pathways. For each figure, different letters indicate significant differences between groups (*p* < 0.05), whereas the same letter indicates no statistically significant difference.

KEGG functional analysis was performed to investigate the effects of different concentrations of DPhP exposure on the gut microbiota function of female zebrafish. At Level 1 functional category (Figure 4A), the relative abundance of functions related to genetic information processing was significantly higher in the High group than in the Control group (*p* = 0.04). At Level 2 functional subcategory (Figure 4B), the relative abundance of energy metabolism function was significantly higher in the Low group than in the Control group (*p* = 0.04). Furthermore, the relative abundance of metabolism of cofactors and vitamins was significantly higher in both the Low (*p* = 0.01) and High (*p* = 0.02) groups compared with the Control group. At the more specific Level 3 pathway level (Figure 4C), the relative abundance of the biosynthesis of amino acids pathway was significantly higher in the High group than in the Control group (*p* = 0.03).

### 3.3. Effects of DPhP Exposure on the Male Gut Microbiota of Zebrafish

#### 3.3.1. Effects of DPhP Exposure on Gut Microbiota Diversity in Male Zebrafish

**Figure 5:**
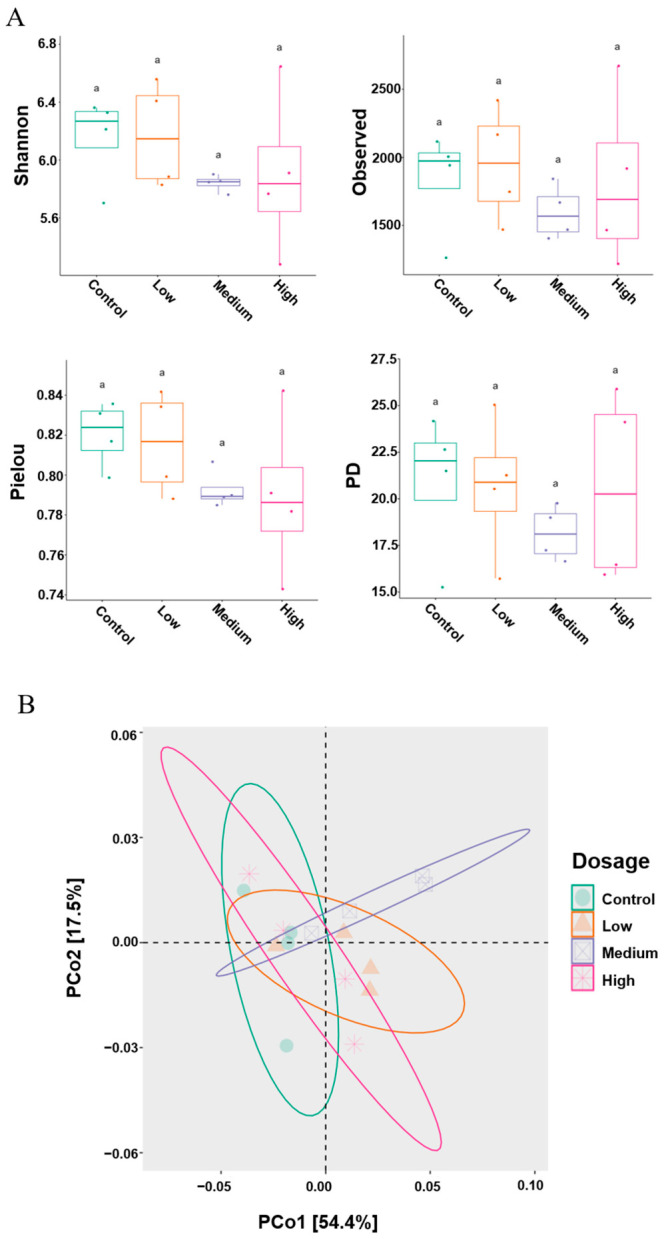
Effects of DPhP exposure on gut microbiota diversity in male zebrafish. (**A**) Alpha diversity indices (Shannon, Observed features, Pielou evenness, and Faith PD) in the control group and under different concentrations of DPhP. Identical letters indicate that the differences are not statistically significant. (**B**) Weighted UniFrac distance-based beta diversity analysis. PCoA plot showing the community structure variation in the gut microbiota in male zebrafish across the control and different DPhP exposure groups.

DPhP exposure did not significantly affect alpha diversity of the gut microbiota in male zebrafish. No significant differences were observed among groups in the Shannon index, Observed features index, Pielou evenness index, or Faith PD index (Figure 5A). Although the Shannon and Observed features indices showed the highest mean values in the Low group and the lowest in the Medium group, and the Pielou and Faith PD indices peaked in the Control group, these trends were not statistically significant. Beta diversity analysis showed that DPhP exposure did not significantly alter the gut microbiota community structure of male zebrafish. Principal coordinate analysis (PCoA) revealed overlapping confidence intervals among groups, with no clear separation in community composition (Figure 5B).

#### 3.3.2. Alterations in Gut Microbiota Composition and Enrichment of Group-Specific Biomarkers in Male Zebrafish Under DPhP Exposure

**Figure 6:**
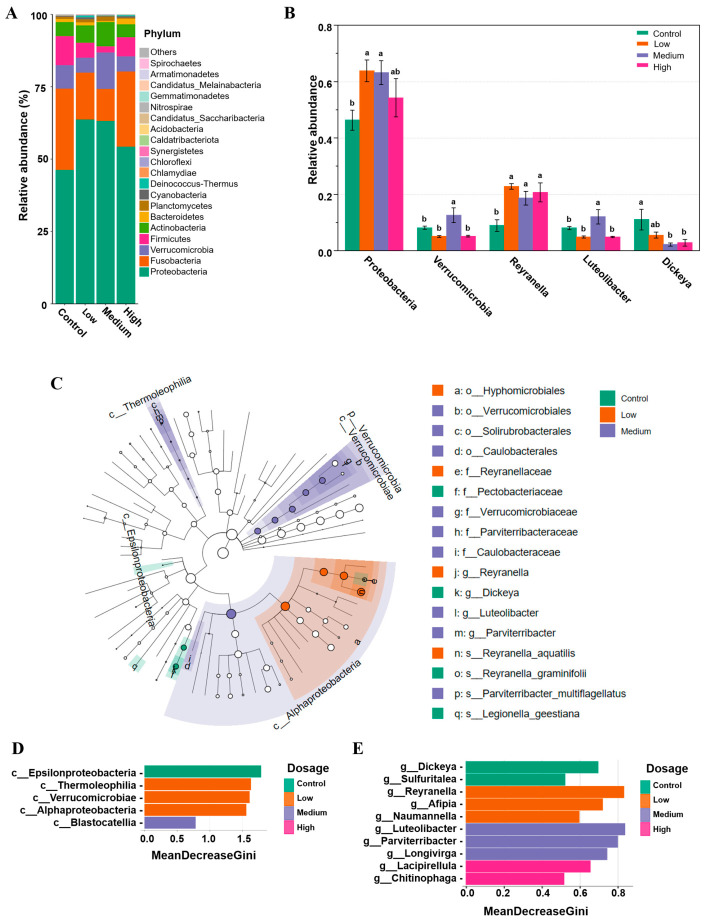
Alterations in gut microbiota composition and enrichment of group-specific biomarkers in male zebrafish under DPhP exposure. (**A**) Relative abundance of gut microbiota at the phylum level in each experimental group. (**B**) Differential relative abundance of gut microbiota at the phylum, class, and genus levels in each experimental group. Different letters indicate significant differences between groups (*p* < 0.05), whereas the same letters indicate no statistically significant difference. (**C**) LEfSe cladogram of the gut microbiota in male zebrafish. Circles radiating from the inside to the outside represent taxonomic levels from kingdom to species. Nodes in different colors indicate taxa significantly enriched in the group corresponding to that color; colorless nodes indicate taxa with no significant differences between groups. Node diameter is proportional to the relative abundance of the taxon. Major taxonomic levels such as phylum and class are labeled directly on the diagram, while other levels are marked with letters, and their specific names are provided in the legend on the right. (**D**) Random forest analysis of the gut microbiota at the class level in each experimental group. (**E**) Random forest analysis of the gut microbiota at the genus level in each experimental group.

At the phylum level (Figure 6A), the gut microbiota of male zebrafish in all groups was dominated by Proteobacteria, Fusobacteria, Verrucomicrobia, Firmicutes, and Actinobacteria, together accounting for 95% of the cumulative relative abundance. However, the relative abundance ranking and compositional patterns of these phyla varied with DPhP concentration. Specifically, the ranking of major phyla from high to low was consistent between the Control and High groups: Proteobacteria > Fusobacteria > Firmicutes > Verrucomicrobia > Actinobacteria. In the Low group, the ranking was Proteobacteria > Fusobacteria > Actinobacteria > Firmicutes > Verrucomicrobia, while in the Medium group it was Proteobacteria > Verrucomicrobia > Fusobacteria > Actinobacteria > Firmicutes. ANOVA (Figure 6B) showed that compared with the Control group, the relative abundance of Proteobacteria was significantly increased in the Low (*p* = 0.02) and Medium (*p* = 0.03) groups, and the relative abundance of Verrucomicrobia was significantly higher in the Medium group than in the Control group (*p* = 0.04).

Combined LEfSe (Figure 6C) and random forest (Figure 6D,E) analyses identified *Dickeya* as a biomarker in the Control group. LEfSe analysis further revealed that Pectobacteriaceae (the family of *Dickeya*) was also significantly enriched in the Control group. Consistently, ANOVA (Figure 6B) confirmed that the relative abundance of *Dickeya* in the Control group was significantly higher than in the Medium (*p* = 0.01) and High (*p* = 0.01) groups. In the Low group, both LEfSe and random forest analyses jointly identified *Reyranella* as a biomarker, and LEfSe analysis showed that this bacterium was significantly enriched from order to species level (*Reyranella aquatilis*). ANOVA indicated that the relative abundance of *Reyranella* in the Low (*p* = 0.002), Medium (*p* = 0.01), and High (*p* = 0.005) groups was significantly increased compared with the Control group. For the Medium group, LEfSe and random forest analyses jointly identified *Luteolibacter* as the biomarker, and LEfSe analysis showed that it was significantly enriched from phylum to genus level in the Medium group. ANOVA further supported that the relative abundance of *Luteolibacter* in the Medium group was significantly higher than in the Control group (*p* = 0.04). No significant biomarker was detected in the High group.

#### 3.3.3. KEGG Functional Analysis of Gut Microbiota in Male Zebrafish Under DPhP Exposure

**Figure 7:**
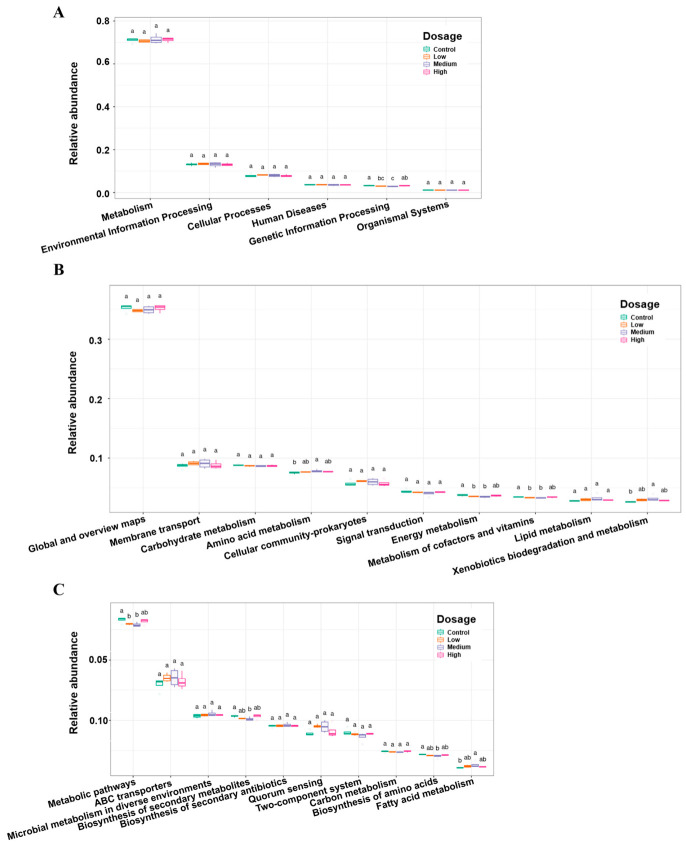
Differential relative abundance of KEGG functional genes in the gut microbiota of male zebrafish under DPhP exposure. (**A**) Level 1 functional categories. (**B**) Level 2 functional subcategories. (**C**) Level 3 functional pathways. For each figure, different letters indicate significant differences between groups (*p* < 0.05), whereas the same letter indicates no statistically significant difference.

KEGG functional analysis revealed that at Level 1 (Figure 7A), compared with the Control group, both the Low (*p* = 0.04) and Medium (*p* = 0.02) groups exhibited significantly reduced relative abundance of the genetic information processing function. At Level 2 (Figure 7B), the relative abundance of amino acid metabolism (*p* = 0.03) and xenobiotics biodegradation and metabolism (*p* = 0.01) functions in the Medium group was significantly higher than that in the Control group. In contrast, the energy metabolism in the Low (*p* = 0.04) and Medium (*p* = 0.03) groups were significantly lower than that in the Control group. Similarly, the metabolism of cofactors and vitamins functions in the Low (*p* = 0.04) and Medium (*p* = 0.04) groups were also significantly lower than that in the Control group. At Level 3 (Figure 7C), the relative abundance of metabolic pathways functions in both the Low (*p* = 0.03) and Medium (*p* = 0.02) groups was significantly lower than that in the Control group. Additionally, the relative abundance of biosynthesis of secondary metabolites (*p* = 0.04) and biosynthesis of amino acids (*p* = 0.008) functions in the Medium group was significantly reduced compared with the Control group, while the relative abundance of fatty acid metabolism function was significantly increased (*p* = 0.04).

## 4. Discussion

The gut microbiota plays an essential role in host metabolism, immune regulation, and response to environmental stressors. Although organophosphate flame retardants have been reported to disrupt the gut microbiome, the specific effects of their metabolites, especially DPhP, remain poorly understood, and sex-dependent responses have not been systematically examined. To address this gap, the present study reveals significant sex-differential responses of the gut microbiota to life-cycle DPhP exposure in zebrafish. Notably, the sex-specific patterns of microbiome alteration observed here align with previously reported male-biased growth inhibition under the same exposure regimen [30]. It should be emphasized that this alignment is correlative, and the present data do not directly establish a causal relationship between gut microbiota changes and growth inhibition. The results showed that after 120 days of exposure, the gut microbiota of female and male zebrafish exhibited distinct response patterns. This sex-specific disruption of the microbiota is consistent with recent reports that organophosphorus flame retardants exert sex-dependent effects on the gut microbiota of organisms [20,21]. Given that the gut microbiota plays a central role in host nutrient absorption, immune regulation, and the metabolism of xenobiotics [42,43], these findings suggest that sex should be considered a critical biological variable that cannot be overlooked when assessing the aquatic health risks of organophosphorus flame retardants and their metabolites.

Multiple organophosphorus flame retardants have been reported to exert sex-dependent effects on the gut microbiota [21,44]. The present study further observed significant differences between female and male zebrafish in terms of gut microbiota diversity, composition, and functional gene responses. While differences in sex hormones [45,46,47] and sex-related immune capacity [48,49] have been suggested in the literature as potential contributing factors, these parameters were not directly examined in the present study; therefore, this interpretation remains preliminary and awaits further validation.

Previous studies have shown that female zebrafish exposed to DPhP (0.8, 3.9, and 35.6 μg/L) throughout their life cycle did not show significant changes in body length or weight [30]. In the present study, we further found that these females exhibited response patterns consistent with environmental stress by reshaping their gut microbiota and increasing the relative abundance of core functional groups. This response did not follow a monotonic dose–response relationship but rather a concentration-driven nonlinear process. At the low concentration (0.8 μg/L), mild stress suppressed the overgrowth of a few dominant bacteria, freeing niche space for others and increasing alpha diversity, particularly evenness [50]. Correspondingly, Tax4Fun2-predicted genes for energy metabolism and for cofactor/vitamin metabolism were both upregulated, which may reflect a metabolic adjustment to meet increased host demand [44,51,52]. At the medium concentration (3.9 μg/L), this apparent compensatory pattern shifted: all alpha diversity indices dropped to their lowest, and beta diversity diverged from the control, indicating that the buffering threshold was exceeded and community restructuring occurred. In the vacated niches, Fusobacteria (mainly *Cetobacterium ceti*) proliferated explosively. *Cetobacterium* is a beneficial anaerobic bacterium that synthesizes vitamin B_12_ and short-chain fatty acids [53,54]. Its enrichment could represent a potentially compensatory response to recruit multifunctional probiotics, which may help stabilize the gut barrier and sustain growth under stress [54,55]. Although we did not measure dietary vitamin B_12_ content or host B_12_ status in this study, the proliferation of *Cetobacterium* may still benefit the host. It is recognized as a core genus in the fish gut microbiota, and its enrichment has been associated with enhanced pathogen resistance and the maintenance of gut ecological stability [56]. However, given that zebrafish have rapid gut transit times and limited hindgut fermentation capacity, the actual nutritional benefit of this enrichment remains speculative and requires further investigation. At the high concentration (35.6 μg/L), *Dickeya* (a potent degrader of complex carbohydrates) became a biomarker, potentially providing carbon skeletons for biosynthesis [57,58]. Consistently, functional genes for genetic information processing, cofactor/vitamin metabolism, and amino acid biosynthesis were all significantly increased, suggesting a potential enhancement of microbial biosynthetic capacity to support basic host functions. In summary, following DPhP exposure, the gut microbiota of female fish underwent a step-wise transition from functional activation and structural remodeling to a state suggestive of enhanced synthetic activity, a shift that may contribute to maintaining host growth stability through microbial functional adjustments. We acknowledge that these functional interpretations are based on Tax4Fun2-predicted pathways rather than direct measurements, and therefore remain hypothetical.

In stark contrast to the response pattern employed by female zebrafish to achieve functional compensation through microbiome remodeling, the gut microbiota of male zebrafish exposed to DPhP exhibited a relatively stable community structure but systemic functional impairment. In terms of community diversity, neither alpha nor beta diversity showed statistically significant changes in male fish. Although alpha diversity did not change significantly, alpha diversity indices showed a downward trend following DPhP exposure, which may indicate a certain degree of negative impact [59]. In terms of community composition, the overall composition of the male zebrafish gut microbiota was similar before and after exposure. However, the relative abundance of Proteobacteria significantly increased after DPhP exposure, and Verrucomicrobia also rose significantly in the medium-concentration group, suggesting that DPhP exposure affected specific phyla in male zebrafish. Studies have shown that an increase in Proteobacteria is often associated with persistent immune responses and chronic inflammation in various host species [60]. Nagalingam et al. [61] induced murine colitis in mice using dextran sodium sulfate (DSS) and found that Verrucomicrobia was present only in the DSS-treated group, suggesting that this phylum is closely associated with the development of colitis in that model. Furthermore, some members of Verrucomicrobia include bacteria that degrade the intestinal mucus layer; an increase in their abundance has been linked to mucus layer degradation and compromised barrier integrity in previous studies [62], suggesting a potential association that warrants further investigation. However, these interpretations are inferential and based on taxonomic associations reported in the literature, as we did not directly measure mucus thickness, intestinal permeability, tight junction protein expression, or inflammatory cytokines in the current study. Therefore, although the overall gut microbiota composition of male fish remained largely unchanged, DPhP exposure may affect the male gut health by altering the relative abundance of specific phyla.

At the functional level, female zebrafish showed significant increases in the relative abundance of functional genes related to energy metabolism and cofactor/vitamin metabolism at the low concentration, as well as genetic information processing and amino acid biosynthesis at the high concentration, reflecting an active, stepwise compensatory response. In contrast, the same functional categories, namely energy metabolism, cofactor/vitamin metabolism, and genetic information processing, were significantly decreased in male zebrafish at low to medium concentrations, indicating systemic functional impairment of the male gut microbiota. However, male fish in the medium concentration group exhibited a distinct pattern: the relative abundance of functional genes involved in amino acid metabolism, xenobiotics biodegradation and metabolism, and fatty acid metabolism increased significantly. The upregulation of xenobiotics biodegradation and metabolism can be interpreted as an adaptive attempt of the gut microbiota to detoxify DPhP. Meanwhile, the increased amino acid metabolism and fatty acid metabolism, together with the suppression of core energy supply and biosynthetic functions (e.g., energy metabolism and amino acid biosynthesis), suggest that the predicted KEGG pathways point toward a potential shift toward catabolic metabolism in the male gut microbiota. However, as these functional changes were predicted by Tax4Fun2 based on 16S rRNA data rather than directly measured, we cannot confirm that the microbiota actually decomposed host-derived amino acids or lipids. Future studies measuring serum amino acid profiles, serum/intestinal lipidomics, or targeted metabolomics of gut contents would be needed to validate this catabolic shift [63,64]. This finding is corroborated by the significantly increased relative abundance of Verrucomicrobia under medium-concentration exposure, which may imply degradation of the intestinal mucus layer. Collectively, these observations point to a potential pathological compensatory mechanism that may occur at the expense of intestinal barrier integrity, though direct evidence for barrier disruption is lacking in the present study. Furthermore, previous studies showed that, compared with the control group, male fish in all exposure groups exhibited decreasing trends in body weight and body length. In the high-concentration exposure group, both body weight and body length were significantly reduced [30]. The causal direction between gut microbiota functional impairment and growth inhibition cannot be conclusively determined from the current data. While we have interpreted the functional impairment of the male gut microbiota as a potential contributor to growth inhibition, it remains equally possible that DPhP-induced growth retardation or associated physiological changes indirectly affect gut microbiota composition and function. Distinguishing between these possibilities would require targeted experiments, such as microbiota transplantation or temporal time-course analyses. This is likely because the impaired core energy-supplying and synthetic functions of the male fish microbiota lead to dual deficits in energy and nutrients, ultimately manifesting as inhibited growth in male fish, a phenotype we previously documented in zebrafish exposed to DPhP under identical conditions. One notable observation is the limited response in the high-concentration male group (35.6 μg/L), where few significant changes were detected in diversity, composition, or predicted function. This could reflect systemic toxicity that alters the host environment and pushes the microbiota toward a new equilibrium, adaptive reorganization of stress-tolerant taxa [65], or hormetic effects [66]. Greater inter-individual variability at this concentration may also have reduced statistical power. The growth inhibition previously observed in males at 35.6 μg/L [30] may represent a host response that influences the microbiota through pathways not captured by diversity indices or functional predictions. Clarifying this pattern will require additional dose points and host physiological measurements.

It is worth noting that several factors could be further explored in future work. The functional interpretations presented here are primarily based on taxonomic composition and predicted pathways, and direct measurements of microbial activity or host physiological responses would provide additional support for these inferences. Similarly, while our data suggest potential shifts in intestinal barrier function, histopathological and permeability evaluations, as well as quantifications of tight junction proteins and pro-inflammatory cytokines, were not measured in the current experiment. These direct biomarkers would be useful to verify our speculations, and the absence of these detections represents a limitation of our study. The absence of water column microbiome data also represents an opportunity for future investigation. While we cannot completely rule out the influence of environmental bacterial uptake, the concentration-dependent and sex-specific patterns observed here suggest that the gut microbiota alterations primarily reflect host-mediated responses to DPhP rather than passive environmental acquisition alone. Furthermore, species-level taxonomic assignments should be considered preliminary given the inherent resolution limits of the V3-V4 amplicon. Lastly, measurements of sex hormones and immune parameters, which were beyond the scope of this investigation, could help clarify the mechanisms underlying the sex-dependent responses observed in this study.

## 5. Conclusions

In conclusion, this study reveals that life-cycle exposure to environmentally relevant concentrations of DPhP induces sex-specific effects on the gut microbiota of zebrafish. Sexually dimorphic responses were observed: females exhibited a dose-dependent functional compensation, whereas males displayed functional impairment without significant diversity changes, suggesting that functional metrics may be more sensitive indicators of pollutant effects than diversity alone. These findings underscore the necessity of considering sex as a critical biological variable in environmental risk assessment of organophosphate flame retardant metabolites. From an ecological perspective, the sex-related functional shifts observed here suggest that chronic DPhP exposure could affect population fitness, highlighting the need for sex-specific considerations in aquatic risk assessment.

## Data Availability

The raw sequencing data have been submitted to the NCBI SRA (submission accession: SUB16302424) and will be publicly available upon publication. The final accession number will be provided at that time.
